# Characterization and population dynamics of germ cells in adult macaque testicular cultures

**DOI:** 10.1371/journal.pone.0218194

**Published:** 2019-06-21

**Authors:** Swati Sharma, Stefan Schlatt, Ans Van Pelt, Nina Neuhaus

**Affiliations:** 1 Center of Reproductive Medicine and Andrology, Institute of Reproductive and Regenerative Biology, Münster, North Rhine-Westphalia, Germany; 2 Center for Reproductive Medicine, Amsterdam UMC Location AMC, University of Amsterdam, Amsterdam, the Netherlands; National Cancer Institute, UNITED STATES

## Abstract

**Background:**

From a biological and clinical perspective, it is imperative to establish primate spermatogonial cultures. Due to limited availability of human testicular tissues, the macaque (*Macaca fascicularis*) was employed as non-human primate model. The aim of this study was to characterize the expression of somatic as well as germ cell markers in testicular tissues and to establish macaque testicular primary cell cultures.

**Materials and methods:**

Characterization of macaque testicular cell population was performed by immunohistochemical analyses for somatic cell markers (SOX9, VIM, SMA) as well as for germ cell markers (UTF1, MAGEA4, VASA). Testicular cells from adult macaque testes (n = 4) were isolated and cultured for 21 days using three stem cell culture media (SSC, PS and SM). An extended marker gene panel (*SOX9*, *VIM*, *ACTA2; UTF1*, *FGFR3*, *MAGEA4*, *BOLL*, *DDX4*) was then employed to assess the changes in gene expression levels and throughout the *in vitro* culture period. Dynamics of the spermatogonial population was further investigated by quantitative analysis of immunofluorescence-labeled MAGEA4-positive cells (n = 3).

**Results:**

RNA expression analyses of cell cultures revealed that parallel to decreasing *SOX9*-expressing Sertoli cells, maintenance of *VIM* and *ACTA2-*expressing somatic cells was observed. Expression levels of germ cell marker genes *UTF1*, *FGFR3* and *MAGEA4* were maintained until day 14 in SSC and SM media. Findings from MAGEA4 immunofluorescence staining corroborate mRNA expression profiling and substantiate the overall maintenance of MAGEA4-positive pre- and early meiotic germ cells until day 14.

**Conclusions:**

Our findings demonstrate maintenance of macaque germ cell subpopulations *in vitro*. This study provides novel perspective and proof that macaques could be used as a research model for establishing *in vitro* germ cell-somatic cell cultures, to identify ideal culture conditions for long-term maintenance of primate germ cell subpopulation *in vitro*.

## Introduction

Spermatogonia present at the basement membrane of the seminiferous tubules are the least differentiated germ cells in adult primate testes [1,2,3]. Previous experimental studies postulate the role of testicular niche factors and Sertoli cells in regulating germ cell dynamics and maintaining testicular homeostasis [4,5,6]. However, the specific mechanisms regulating self-renewal and differentiation of spermatogonia in primates are not yet completely understood [7,8,9,10,11]. For understanding spermatogonial regulation in primates, establishing primate-specific *in vitro* approaches for co-culturing testicular germ cells and somatic cells isolated from adult testes are considered highly valuable.

Most of the previous culture studies using adult human [10,12,13,14,15,16,17,18] and non-human primate [19] testicular tissues, showed maintenance of spermatogonia in culture for limited time. Similarly, several attempts to isolate and culture mammalian Sertoli cells *in vitro* were reported to investigate their regulatory, biological and secretory function [20,21,22,23,24,25,26,27,28,29,30]. However, it was challenging to culture Sertoli cells from adult primates as they are known to rarely divide *in vivo* [31] or *in vitro* [22,23].

Due to limited or non-availability of normal adult human testicular material for culture, healthy adult non-human primates are used as an alternative model for biological and pre-clinical research. Cynomolgus and rhesus monkey species belonging to the old world primate family *Cercopithecidae*, share close phylogenetic relationship, similar testicular developmental patterns, spermatogenic stages (12 stages) [5] and stem cell progenitor systems with humans [8,32,33,34,35,36]. Therefore, macaques are considered a highly relevant non-human primate research model to study male reproductive physiology and testicular function, as the findings can potentially be extrapolated to humans. In the current study, we employ *Macaca fascicularis* as a research model. We aim to characterize cellular sub-populations in adult macaque testis, establish macaque testicular cell cultures and investigate the survival and maintenance of macaque germ cells *in vitro*. Furthermore, we aim to understand the population dynamics of macaque spermatogonia in culture.

## Materials and methods

### Adult macaque testicular donors, tissue retrieval and processing

In Germany the license to maintain and breed macaques allows under governmental approval (§4 German Animal Protection Law) the right to sacrifice animals when their organs and tissues are dissected and processed to perform research studies. Animals are killed by an injection of an overdose of anesthetics. Here, we killed the monkeys by an i.v. infusion of a lethal dose of pentobarbital. Independent veterinarians confirmed the death of the monkeys prior to necropsy. Both Centers providing monkey tissue (CeRA, Covance Inc.) hold governmental licenses to maintain and breed cynomolgus monkeys. In order to target the 3R principles (reduction, replacement, refinement) and to minimize the number of animals used in animal studies sharing of organs is recommended. The monkeys used in this study were primarily serving as untreated control animals in toxicology studies. The dissection of reproductive organs enabled additional studies unrelated to the aim of the primary study.

The license number for CeRA approval is #39.32.7.1 provided by the veterinary office of the City of Münster on Sep 11, 2013. The use of tissue from animals is annually registered under this license. On necropsy the testes were obtained to enable the *in vitro* studies Prior to sacrifice the monkeys were housed in an AAALAC certified animal facility including oversight by an IACUC committee and standard operating procedures for all aspects in animal maintenance. Macaques are group housed (3–5 monkeys) in pens (1.6 m x 1.6 m x 2.66 m) with cage mates being constantly present for enrichment. On a daily basis the health status of each individual monkey were recorded. Feeding pellets were distributed on the floor with seeds added to the bedding (wood chips) which encourages foraging. Pieces of wood (10 cm x 10 cm x 10 cm) are supplied for chewing. On a weekly basis enrichment devices (cardboard box filled with hay and seeds, pipes filled with hay and seeds and small barrels filled with hay and seeds) are provided for entertainment. The enrichment toys are rotated weekly and the enrichment strategies are documented. The cages were designed for environmental enrichment by creating multi-level housing conditions like balconies and visual barriers In accordance to the AAALAC certification the procedures guaranteed effective maintenance of their social, psychological and physical health under caging conditions.

Testes from 4 adult macaque monkeys (*M*. *fascicularis*) were used for the cell culture study and testis samples from remaining monkeys were used for histological analysis (Table 1). Serum samples were collected for testosterone measurements. During necropsy, the tunica albuginea was removed, and testes were dissected. Testicular tissue was placed in chilled Minimum Essential Medium-alpha (Mem-α) medium (22561–021, Life Technologies GmbH, Gibco, Darmstadt, Germany). Additional tissue fragments separated as pre-culture control were fixed in Bouin’s solution for characterization of macaque testicular markers by immunohistochemistry analysis.

**Table 1:** Parameters of adult macaque testicular donors.

Macaque number	Age	Body weight (kg)	Testosterone (nmol/l)	Total testicular weight (g)
1	9y 8m	7.5	18	41.4
2	6y 5m	9.2	39.6	52.9
3	9y 8m	10.7	55.8	65.1
4	13y 5m	13	48.7	64
5	8y 8m	11.9	-	82.4
6	10y 8m	8.4	-	56.6
7	8y 2m	7.7	-	48.3
Mean	9y 6.2m (±0.7y 4.2m)	9.6 (±0.14)	40.5 (±16.4)	58.6 (±4.8)

Reference values: Age (>4 years (y) 3 months (m)), body weight (6.4±1.7 kg), testosterone (20.8–40 nmol/l), total testicular weight (>33.7 g). [35,36]

### Testosterone measurements

Serum testosterone of donor monkeys was assessed using an established radioimmunoassay (RIA) protocol [37,38]. An iodinated tracer (testosterone-3-CM-histamine) was used employing the chloramine-T/sodium meta-bisulfite method and an antiserum raised in rabbit against testosterone-3.

### Identification and validation of testicular germ cell and somatic cell markers by immunohistochemistry

Macaque testicular sections from different age groups (neonatal and adult) were subjected to immuno-histochemical analysis. Neonatal macaque testicular sections were acquired from previous ethically approved research study and adult macaque testicular sections were acquired from the current study. After de-paraffinization and rehydration, antigen retrieval was performed using sodium citrate buffer (pH 6). Testicular sections were kept on ice, subsequently washed with TBS and incubated with 3% (v / v) H_2_O_2_ (hydrogen peroxidase) for 15 minutes at room temperature. Non-specific binding sites in the sections were blocked by incubation in the blocking buffer containing 25% chicken / goat serum with 0.5% (w / v) BSA in TBS for 30 minutes. Sections were incubated overnight at 4°C with primary antibodies directed against SRY (sex determining region Y)-box 9 (SOX9), vimentin (VIM), actin-alpha 2-smooth muscle (ACTA2) also known as α-SMA, undifferentiated transcription factor (UTF1), melanoma antigen family A 4 (MAGEA4) and DEAD-box helicase 4 (DDX4) also known as VASA (S2 Table). Sections were incubated with biotinylated secondary antibodies chicken anti-mouse and chicken anti-rabbit and subsequently with streptavidin horse-radish peroxidase (HRP) for 45 minutes. Staining with chromogen 3–3´-diaminobenzidine was performed to visualize protein expression patterns. Sections were subsequently counterstained with hematoxylin, dehydrated and incubated in apiclear for 20 minutes, before mounting. Isotype IgG controls were included as negative controls for all the primary antibodies. Stained sections were visualized using an Olympus BX61 microscope connected to a Retiga 400R camera (Olympus, Melville, NY, USA). Images were captured for analysis using Cellsens imaging software (Olympus, Melville, NY, USA).

### Isolation and co-culture of testicular germ cells and somatic cells

Seminiferous tubules were dissected into smaller tubular fragments for enzymatic digestion and isolation of spermatogonia. Two step enzymatic digestion of testicular tissue was performed according to the previously described protocol [12], using spermatogonial stem cell (SSC) medium (containing Mem-α, 10% FCS and 1% Pen Strep). DNase1 (Sigma DN25) and trypsin (Gibco27250-018) were used in the first step and collagenase IA (Sigma C9891) was used in the second step employing HBSS (Hank’s balanced salt solution) (Gibco, Lot no.1532349). Cell suspensions were subjected to trypan blue staining and viable cells were counted using the Neubauer chamber. Absolute cell numbers were calculated using the following formula: Mean cell number x volume of cell suspension x 2 (dilution factor trypan blue) x 10,000 (chamber volume).

SSC, Stemmacs (SM) (Miltenyi Biotec, Cat number: 130-107-086, Lot number: 5150618252) and Pluristem (PS) SCM130 (Millipore, Lot number: 2701329) media were used to culture spermatogonia after isolation. The three distinct stem cell culture media were selected particularly to compare the effect of different culture components on survival and maturation of germ cells *in vitro*. SSC media has been previously used to establish and maintain human and marmoset SSC cultures [12,19], thus it served as a control culture condition with published evidence of its efficiency in the two different primate species. To date, feeder free culture conditions commonly used for pluripotent stem cell cultures have not been assessed for culturing primate spermatogonia. Therefore xeno-free SM and PS media commonly used for maintenance and expansion of ES and IPS cells were selected. The two culture media used in combination with cell attachment matrices like matrigel, are known to preserve phenotype, functionality and stem cell potential for long term during culture.

The three media were sterilized using a 0.2-micron filter. SM medium was prepared by mixing Stemmacs iPS-Brew XF medium with Stemmacs iPS-Brew supplement (Lot no: 5150707125). Uncoated 6-well plates were used to culture cells in SSC media as previously described [19]. Pre-aliquoted matrigel (Corning, Cat no.354277, Lot no.5131013) stored at -20°C, was thawed at 4°C. Thawed matrigel was diluted in the ratio of 1:20 with DMEM/F12 media. Diluted matrigel (6 ml per plate i.e. 1 ml per well) was used to coat one 6 well plate (Costar 3516, Corning) using a glass pipette. Freshly coated plates were incubated at room temperature in the hood for at least 1 hour before use. Remaining liquid was aspirated from coated plates just before use; culture medium was added immediately to wells. Matrigel coated plates were thereafter used for seeding cells in SM or PS media. Antibiotic-Antimycotic (Thermo Fisher Scientific, Cat no. 15240062) at a concentration of 20μg/ml, was added to the cultures to prevent microbial growth.

A fraction of cells at day 0 was collected as pre-culture control sample and snap-frozen for mRNA analysis. Approximately 1.2 million cells were seeded per well in 6-well culture plates containing the three media, respectively. After plating, cultured cells were documented microscopically. Subsequently, supernatant (SN) and attached (AT) cell fractions were separated, according to the previously published testicular cell separation and culture approach for human and marmoset cell suspension [12,19]. AT cell cultures were incubated at 35°C and 5% CO_2_ and cultured for 21 days. Every third day, one-third of the media was replaced throughout the culture period. During the three-week culture, cells in the supernatant of the attached (AT) cultures were collected at days 7, 14 and 21. Cells were snap-frozen in liquid nitrogen for gene expression analysis.

### Gene expression analysis of cultured cells

For RNA isolation from snap-frozen testicular fragments and cultured cells, RNAeasy Plusmicrokit (74004, Qiagen, Germany) was employed. cDNA was synthesized by iScript cDNA synthesis kit using 100 ng RNA (I70-8890, BioRad Laboratories GmbH, Germany). Dilution (1:2) of cDNA was performed using RNase free water (129115, Qiagen, Germany). TaqMan based real-time quantitative PCR was performed in reaction volumes of 15 μl (Applied Biosystems) with somatic cell and germ cell specific primers for *M*. *fascicularis* as listed in S1 Table (TaqMan gene expression Master Mix 7.5 μl (4369016, Applied Biosystems), primers 700 nM, water 5.25 μl). A StepOne plus cycler was employed to run qPCR assays. qPCR data analysis was performed by StepOne software 2.2. Screening of 4 reference genes *WT1*, *NONO*, *RPL13A* and *GAPDH* was performed for macaque testicular samples in a previous study [39]. *GAPDH* gene showed stable patterns and was therefore selected as reference gene for normalization of results. Results are represented as 2^−ΔCt^ values [40,41]. Expression of 6 pre-characterized (*FGFR3*, *MAGEA4*, *BOLL*, *DDX4*, *SOX9* and *ACTA2*) [39] macaque-specific primers was validated and additionally, 2 new (*UTF1*, *VIM*) markers were characterized in pre-culture control samples collected at day 0. The eight characterized germ cell and somatic cell marker genes were subsequently employed to assess macaque somatic cell-germ cell dynamics *in vitro*.

### Immunofluorescence analysis of cultured cells

Macaque testicular cells (25000 cells per well) were cultured in 8-well chamber slides (354108, Becton Dickinson GmbH, Germany). Cultured cells were fixed at day 7, 14 and 21 with 4% paraformaldehyde for 30 minutes. Thereafter, PBS was added to each well and chamber slides were covered with parafilm and stored at 4°C prior to analysis. Immunofluorescence stainings of cultured cells were performed for spermatogonial marker Melanoma antigen family A 4 (MAGEA4) and DEAD-box helicase 4 (DDX4) also known as VASA. Cells were first permeabilized with 1% TritonX/TBS and then blocked in PBS containing 25% goat serum, for 30 minutes each. Thereafter, incubation with the primary antibody directed against MAGEA4 and VASA in blocking medium (25% goat serum in PBS) was performed overnight at 4°C (S2 Table). After overnight incubation, cells were washed with 0.1% Tween-20 in TBS at room temperature and incubated for 1 hour with conjugated goat-anti-mouse 488 and donkey-anti-rabbit CyTM3 secondary antibody (S2 Table) in TBS at a 1:100 dilution. Subsequently, cells were washed with 0.1% Tween-20 in TBS. Cells were counter stained with DAPI and embedded in Paramount aqueous mounting medium (S3025, Dako, Germany). Stained cells and negative controls were evaluated using an Olympus BX61 microscope connected to a Retiga 400R camera (Olympus, Melville, NY, USA). Images were captured for analysis using Cellsens imaging software (Olympus, Melville, NY, USA).

### Quantification of MAGEA4-positive cultured testicular cells and clusters

Quantification of MAGEA4-positive cells was performed, for testicular cells cultured in duplicate wells from 3 adult macaque monkeys until day 7, 14 and 21 for all three culture conditions (SSC, PS and SM). Clusters were defined as two or more cells closely connected as described previously [33]. Singlets (1c), duplets (2c), triplets (3c), quadruplets (4c), quintuplets (5c), small clusters of 5–10 cells (sc) and big cell clusters with more than 10 cells (bc) were quantified in duplicate wells for each condition. The mean values of relative percentage of cells and clusters calculated with respect to day 7 for 2 monkeys were plotted in graphs for each time point and the three culture conditions.

### Statistical analysis

Statistically significant differences between relative mRNA expression levels for different genes and time points in all three culture conditions were evaluated using non-parametric Kruskal-Wallis test and Dunn’s multiple comparison test. Statistically significant changes in MAGEA4-positive quantification data were evaluated using one-way ANOVA and post-hoc Tukey test. Significant differences are indicated by single asterisk (*) for P<0.05 and by double asterisks (**) for P<0.02. Graph Pad Prism5 (Graph Pad software version 5) was used for all the statistical analysis.

## Results

### Characterization and validation of testicular somatic and germ cell markers in macaques

**Fig 1:**
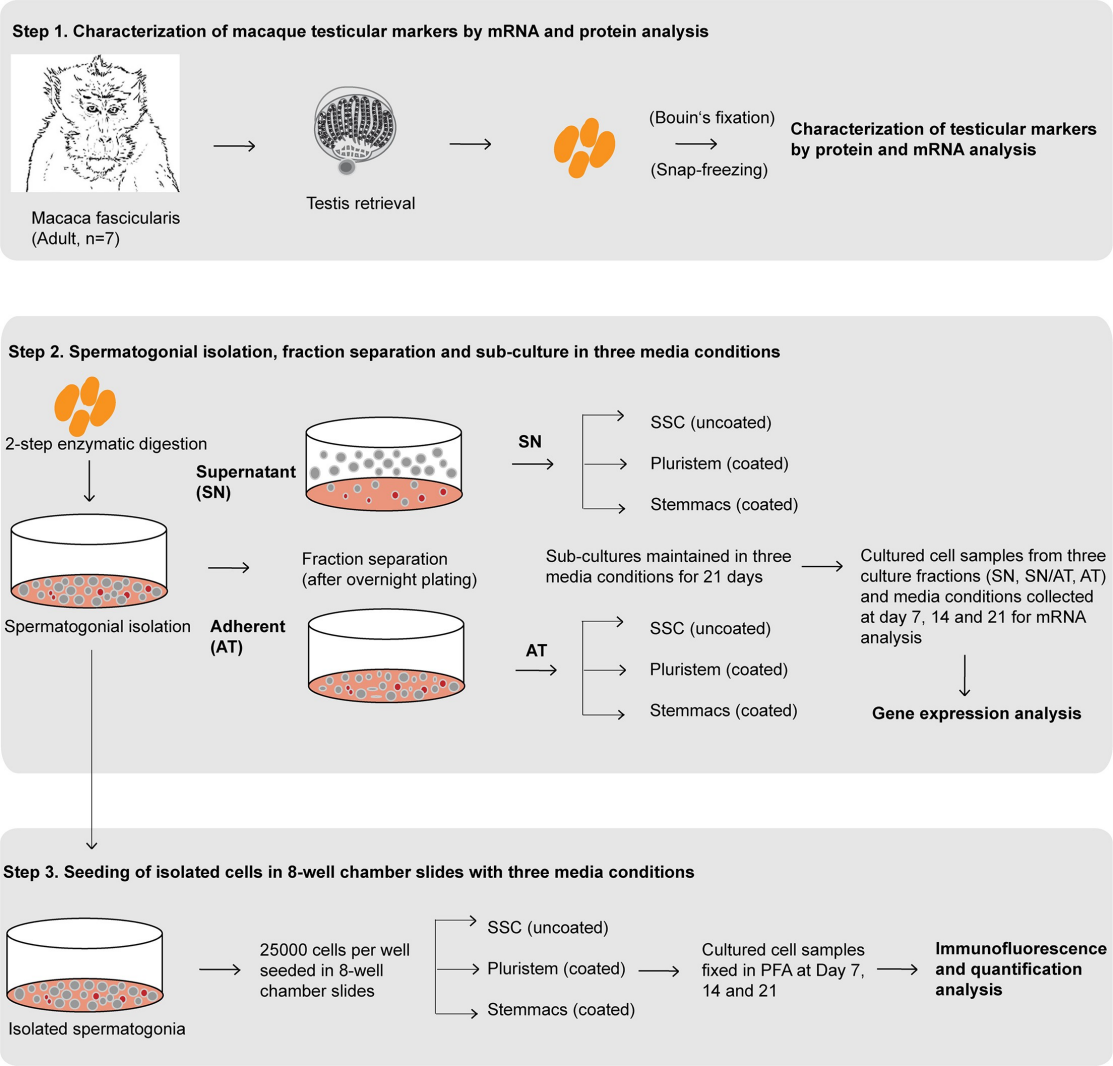
Study design demonstrating three-step experimental scheme. Step 1: Characterization of macaque testicular markers by mRNA and protein analysis. Step 2: Spermatogonial isolation, separation of supernatant (SN) and adherent (AT) fractions and sub-culture in three media conditions (Spermatogonial stem cell (SSC), Stemmacs (SM) and Pluristem (PS) media). Gene expression analysis of cultured cells collected at day 7, 14 and 21. Step 3: Seeding of isolated spermatogonia (25,000 cells per well) in 8-well chamber slides with three media conditions. Fixation of cells cultured in three media conditions in 4% Para-formaldehyde (PFA) at day 7, 14 and 21. Immunofluorescence analysis for expression of germ cell markers MAGEA4 and VASA *in vitro* and quantification of cells and clusters expressing MAGEA4 *in vitro* during culture.

**Fig 2:**
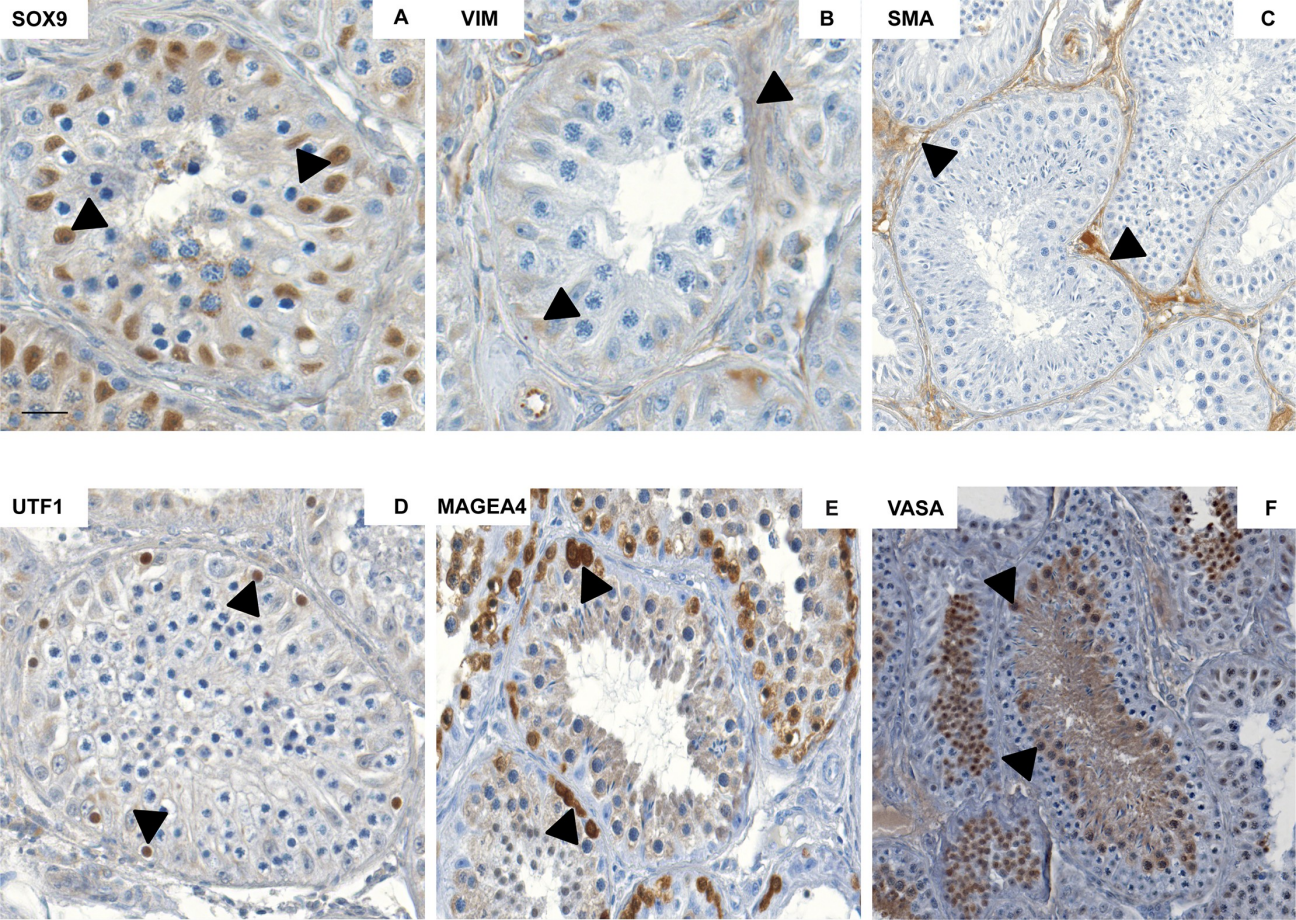
Characterization of somatic cell and germ cell markers in adult macaque testicular sections. Representative images in the micrographs show strong expression for somatic cell markers SOX9 (A), VIM (B) and SMA (C) in adult macaque testicular sections. Intense expression of germ cell markers UTF1 (D), MAGEA4 (E) and VASA (F) was observed in adult macaque testicular sections in representative images in the lower panel. Black arrows in the micrographs indicate marker-specific localization. Scale bar in A, B, D and E indicates 50 μm, and in C and F indicates 100μm.

As depicted in the study design (Fig 1), characterization of testicular cell population in macaques was performed by subjecting neonatal and adult macaque testicular sections to immuno-histochemical (IHC) evaluation. For SOX9, strong nuclear expression was detected in Sertoli cells of neonatal (S1A Fig) and adult testicular sections (Fig 2A). Vimentin (VIM) was detected in the somatic cell population including Sertoli, peritubular as well as interstitial cells in tissue sections of neonatal (S1B Fig) and adult testes (Fig 2B). In contrast to these two markers, expression of α-SMA was dependent on differentiation status. In neonatal testes (S1C Fig), a strong staining was detected only in blood endothelial cells of blood vessels, whereas also a cytoplasmic expression in peritubular myoid cells was detected in adult testes (Fig 2C). Regarding the expression pattern of the spermatogonial marker UTF1, intense nuclear staining was detected in the majority of germ cells in neonatal testicular sections (S1D Fig) whereas only very few UTF1 positively stained cells were detected in adult macaque testicular sections (Fig 2D). For MAGEA4, spermatogonial cell populations representing the undifferentiated germ cells in both neonatal (S1E Fig) and adult sections show strong cytoplasmic expression for MAGEA4, whereas spermatocytes showed weak MAGEA4 staining in adult macaque testicular sections (Fig 2E). Cytoplasmic expression pattern for VASA was comparable for all germ cell types in neonatal (S1F Fig) and adult testicular sections (Fig 2F).

### Comparing morphology of macaque testicular cells in culture

To assess dynamics of macaque spermatogonia *in vitro*, following overnight plating SN and AT cells were separately seeded and population dynamics from both cultures was determined. Primary testicular cultures contained both cells in suspension and attached cells. After separation, morphology of cells observed in the SN fraction included small and round shaped single cells, clusters of duplets, triplets, small sized clusters and clusters of bigger sizes (S2A Fig). Cell population observed in AT cultures included mostly flattened elongated irregular shaped, fibroblastic-like cells attached to the culture dish (S2B Fig).

### Identification of testicular somatic cell and germ cell markers and expression analysis of cultured testicular cells

**Fig 3:**
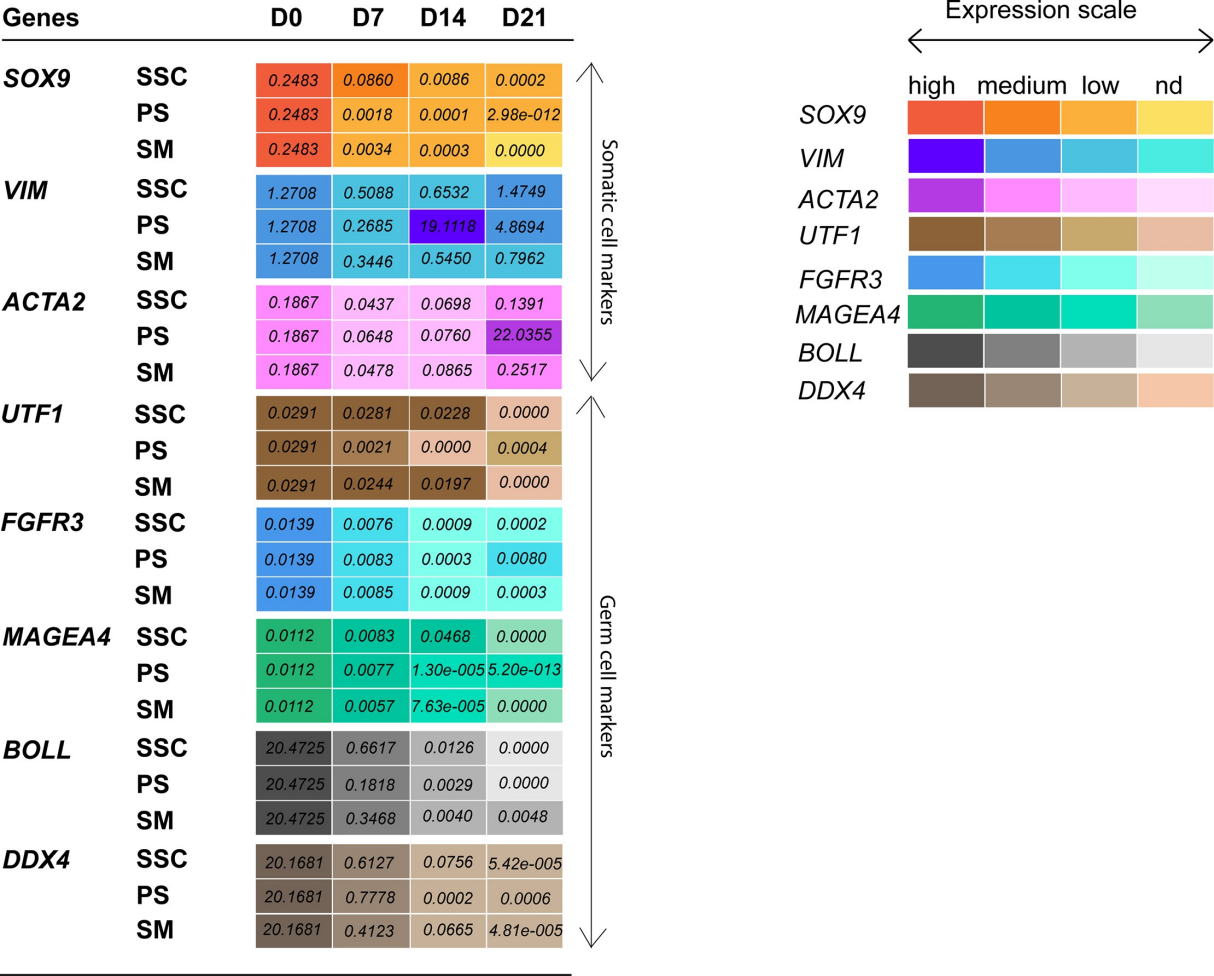
Heat map showing gene expression levels (2^ delta Ct values) of somatic marker genes (*SOX9*, *VIM* and *ACTA2*) and germ cell marker genes (*UTF1*, *FGFR3*, *MAGEA4*, *BOLL* and *DDX4*) relative to reference gene *GAPDH* of macaque testicular cells cultured in SSC, PS and SM media conditions for 21 days. Expression scale for each marker gene represents relative gene expression levels from high to medium, low and non-detected (nd). Each box represents mean values calculated from cultured testicular cells of 4 macaque monkey samples.

Expression of somatic cell specific markers *SOX9*, *VIM* and *ACTA2* (*α-SMA*) was characterized in testicular cell samples collected at culture days 0, 7, 14 and 21. Results (Fig 3, S3 Fig) show a trend of decreasing *SOX9* expression throughout the culture period in all three media conditions. Compared to day 0, significantly reduced *SOX9* expression was observed at day 14 and 21 in PS cultures and at day 21 in SM cultures. *VIM* and *ACTA2* expression were maintained throughout the culture period in all three culture conditions. In addition, expression of undifferentiated (*UTF1*, *FGFR3*), differentiating (*MAGEA4*) and more advanced germ cell subpopulations (*BOLL*, *DDX4*) was characterized (Fig 3, S4 Fig). Expression levels of spermatogonial marker *UTF1* were maintained until day 14 in SSC and SM media. However, significantly reduced expression of *UTF1* was observed at day 14 and 21 in PS cultures. Expression levels of *FGFR3* were maintained and only significantly reduced at day 21 in SSC media. Expression of pre- and early meiotic germ cell marker *MAGEA4* was maintained until day 14 in all three cultures, a significantly reduced expression was observed at day 21 in SSC media. Irrespective of the culture conditions and compared to day 0 a significant decrease in mRNA expression levels for differentiating germ cell markers *BOLL* and *DDX4* was observed.

### Germ cell identification by immunofluorescence analysis

**Fig 4:**
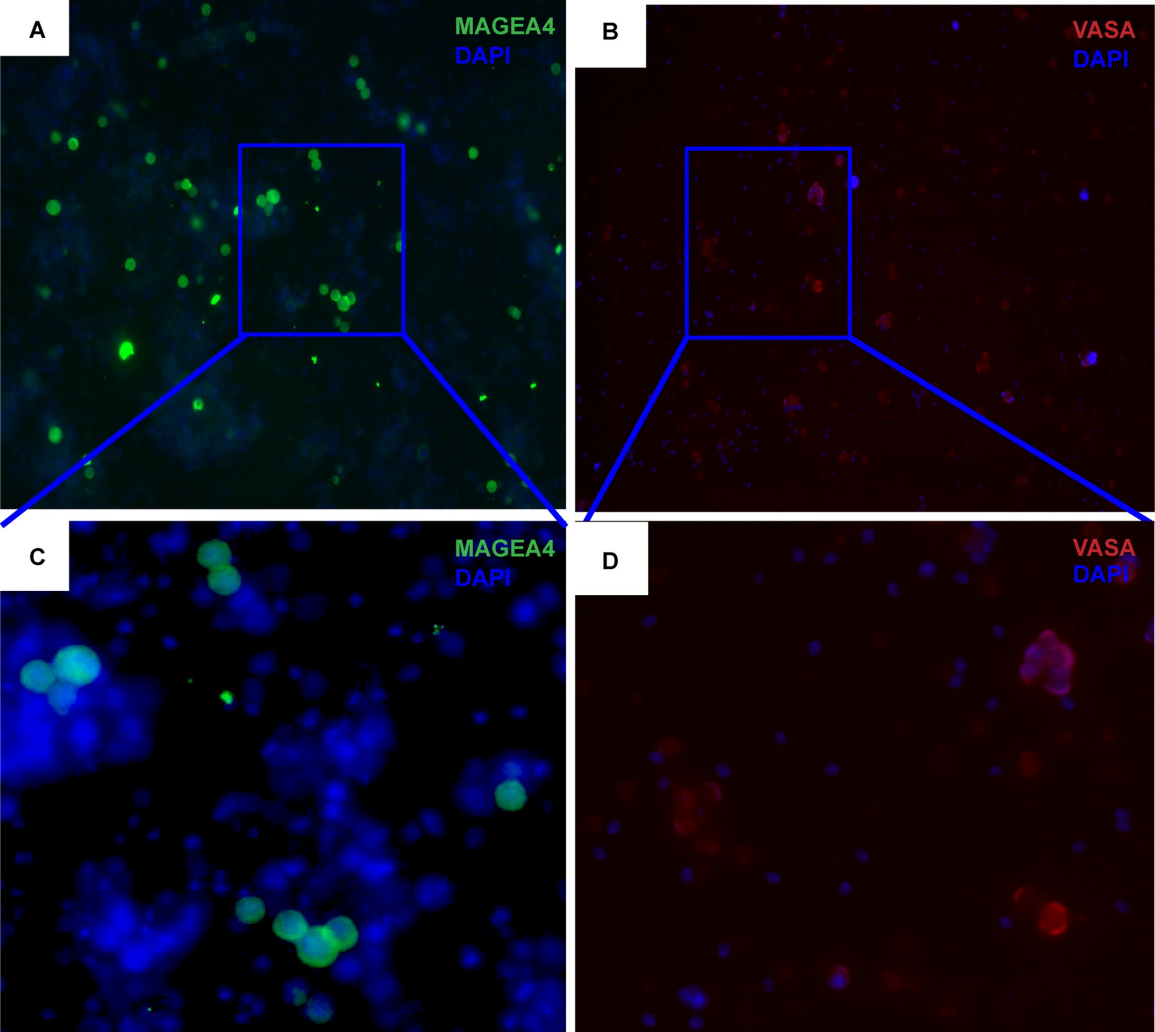
Adult macaque testicular cells cultured in SSC, PS and SM culture conditions were analyzed for expression of the germ cell markers MAGEA4 and VASA *in vitro*. Representative micrographs show cultured cells at day 14 exhibiting strong expression for gem cell markers MAGEA4 (A) and VASA (B) as shown (20x; with Dapi). Highlighted MAGEA4 (C) and VASA (D) labeled single cells and clusters of various sizes are shown (40x; with Dapi).

Cultured cells were analyzed by germ cell markers to localize germ cell sub-populations. Cultured cells were subjected to MAGEA4 (Fig 4A and 4C) and VASA (Fig 4B and 4D) immunofluorescence staining to localize the pre-, early and late spermatogonial and meiotic germ cell populations. These cells were mostly small, round and compact in shape and the size varied based on the maturation state of the cells.

### Quantification of MAGEA4 expressing cultured cells

**Fig 5:**
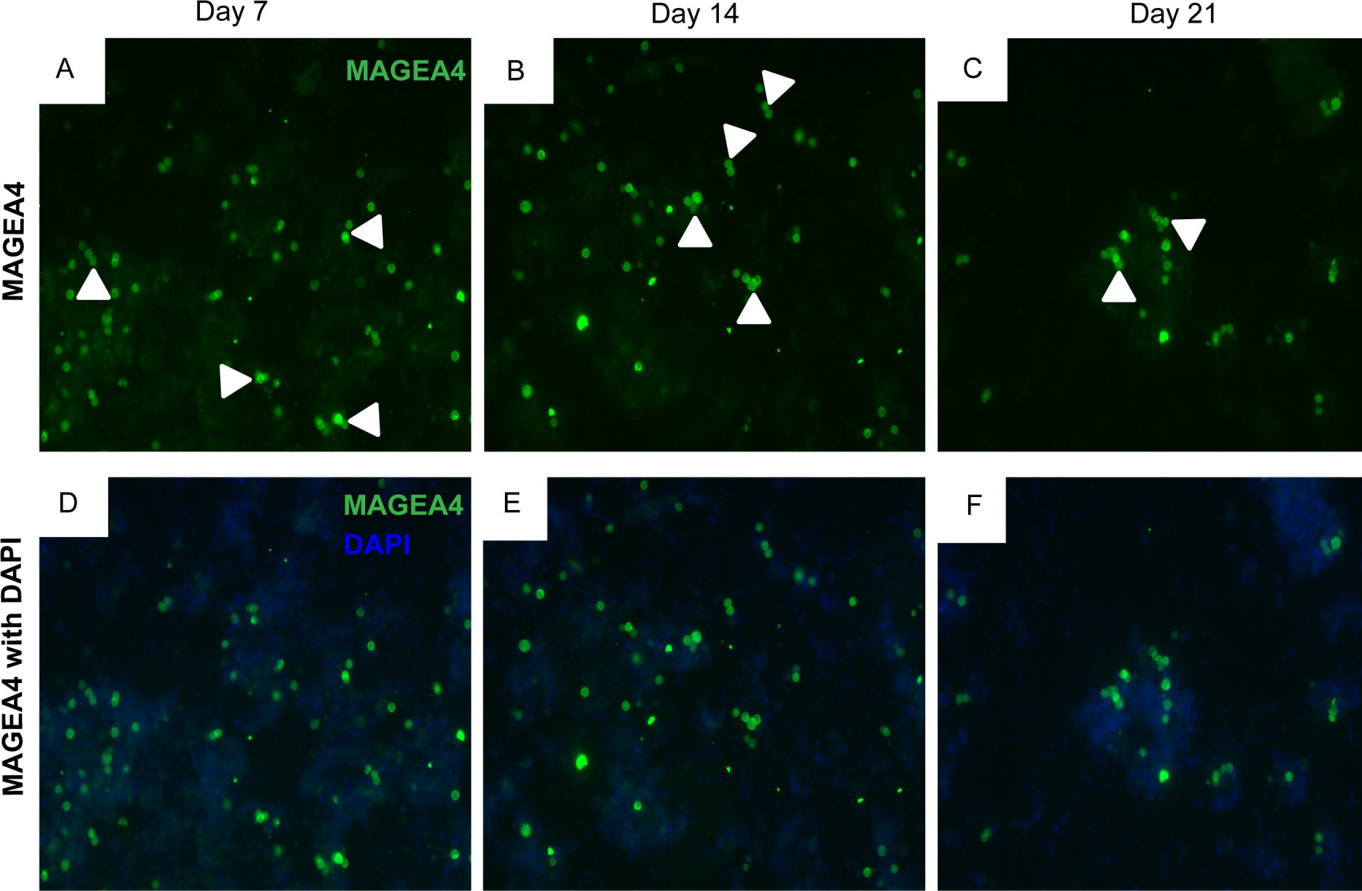
Adult macaque testicular cells cultured in SSC, PS and SM culture conditions were analyzed for expression of the germ cell marker MAGEA4 *in vitro*. Representative micrographs show cultured cells at day 7, 14 and 21 exhibiting strong expression for spermatogonial marker MAGEA4 as shown in A, B, C (20x; without DAPI) and D, E, F (20x; with DAPI). Cells cultured for 7, 14 and 21 days showing intense expression for MAGEA4 were quantified for each culture condition. Singlets (1c), duplets (2c), triplets (3c), quadruplets (4c), quintuplets (5c), small clusters (sc) of 5–10 cells and big clusters (bc) of >10 cells, as shown by white arrowheads were quantified for each culture condition and time point.

Immunofluorescent labeled germ cells (singlets, duplets, triplets, quadruplets, quintuplets, small and big clusters) expressing MAGEA4 were observed in cells cultured in all the three media conditions throughout the culture period (Fig 5A–5C). As shown in the images (Fig 5D–5F), cells expressing MAGEA4 were a small proportion or sub-population of the total testicular cell population present in the culture.

**Fig 6:**
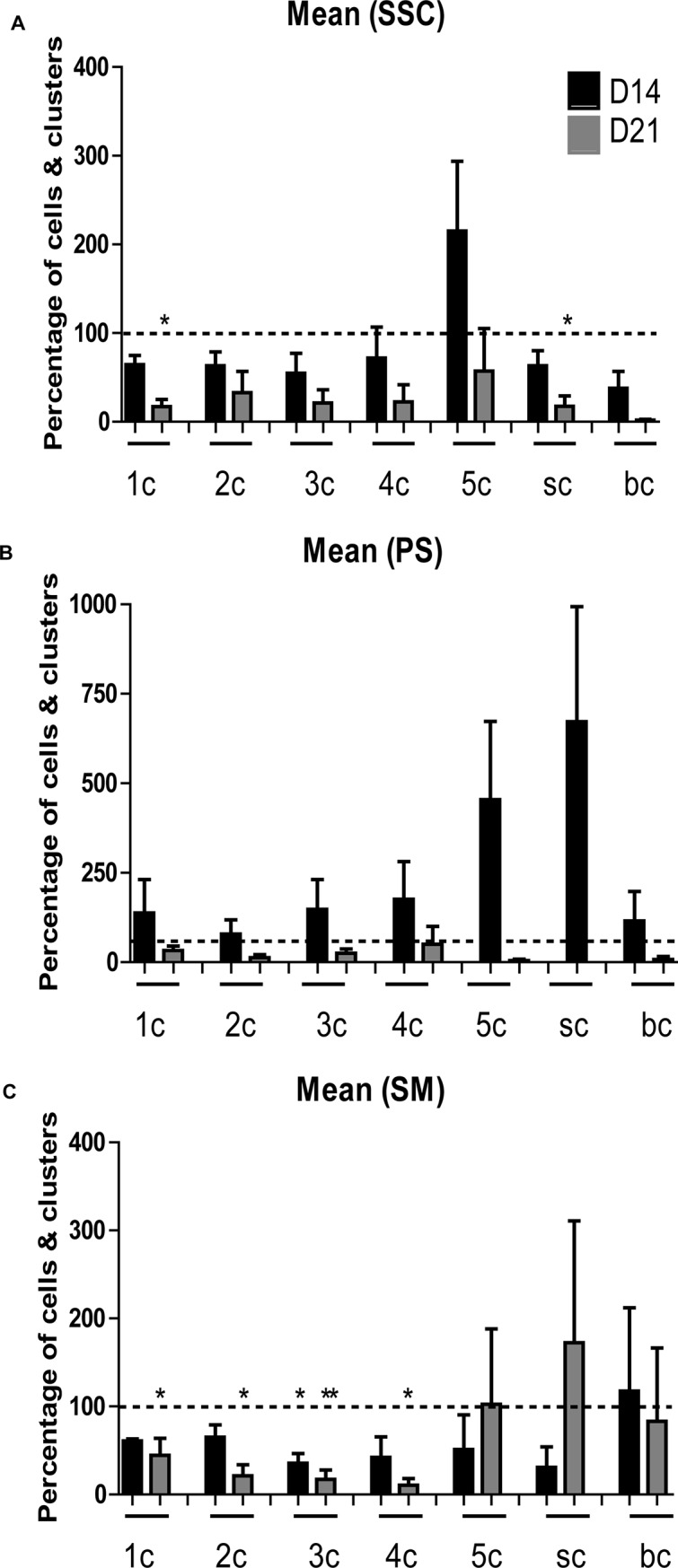
**Graphs represent relative percentage of cells and clusters (ranging from 1 cell (1c), 2 cell (2c), 3 cell (3c), 4 cell (4c), small clusters (sc) of 5–10 cells to big clusters (bc) of >10 cells) expressing the germ cell marker MAGEA4 *in vitro* at day 14 and 21 with respect to day 7, in adult macaque testicular cells cultured in three different media i.e. SSC (A), PS (B) and SM (C) media.** Dotted line indicates the reference value representing the percentage of cells and clusters expressing MAGEA4 at day 7. One-way ANOVA and tukey‘s multiple comparison test was used to calculate statistical significance. Values were considered statistically significant if P<0.05(*) or if P<0.02(**).

The effect of *in vitro* culture for each time point and three media conditions was assessed by quantification of MAGEA4-positive cells and clusters (Fig 6A–6C). The mean percentage values at day 14 and 21 with respect to day 7 for 2 monkeys were calculated. No significant increase in relative percentage of MAGEA4-positive cells and clusters was observed for any time point and culture condition. Percentage of duplets, triplets, quadruplets, quintuplets and big clusters in SSC cultures; percentage of singlets to big clusters in PS cultures and percentage of quintuplets, small and big clusters was maintained in SM cultures. A significant decrease in percentage of triplets in SM culture was observed at day 14 compared to day 7. Whereas, a significant decrease in percentage of singlets and small clusters in SSC and percentage of singlets, duplets, triplets and quadruplets in SM culture at day 21 compared to day 7 was observed. In general, regardless of the culture conditions no significant changes were observed in overall population dynamics of cultured cells overtime.

## Discussion

Here, for the first time we characterized adult macaque testicular sub-populations, and thereafter isolated and cultured testicular cells from adult macaque testes (*Macaca fascicularis)*. Effect of three distinct culture media was analyzed by assessing the change in germ cell-somatic cell expression levels *in vitro* during culture. Differences in depletion and enrichment patterns of specific germ cell and somatic cell sub-populations during culture were detected. Expression analysis using eight characterized marker genes indicates that in accordance with the selective depletion of Sertoli cells amongst the somatic cell sub-populations, a relative depletion of germ cell (undifferentiated, pre- and meiotic and differentiating) sub-populations was observed overtime. The changing germ cell dynamics during culture further confirms the maintenance of pre- and early meiotic germ cells until day 14 in culture.

For the first time we report localization of two germ cell markers (UTF1 and MAGEA4) and two testicular somatic cell markers (VIM and SOX9) in adult macaque testicular sections. Localization of UTF1 and MAGEA4 was comparable to the patterns reported in human [12] and marmoset [19]. Localization of VASA was comparable to the patterns reported in human [12,42], marmoset [19], and macaque [43] testicular sections and cell samples. The characterization of somatic markers and comparable localization of germ cell markers in primate species substantially validates the use of macaques as a suitable research model for understanding primate spermatogonial biology and the use of these markers to study germ cell-somatic cell dynamics *in vitro*.

Expression analysis of cultured cells for Sertoli cell marker gene, *SOX9* in SSC media shows non-significant but reduced expression levels overtime, which indicates a trend of decrease in the number of Sertoli cells in SSC media during culture. However, significantly diminished *SOX9* expression levels were detected in PS and SM cultures at day 21, which demonstrates significant depletion of Sertoli cells in the two culture conditions. No significant change in expression levels for two somatic cell marker genes, *VIM* and *ACTA2* were detected in the three cultures overtime; which is comparable to the previously reported findings in marmosets [19]. This indicates that irrespective of the culture media used, *VIM* and *ACTA2* sub-populations were maintained in all three macaque testicular cultures overtime. In contrast, expression levels of undifferentiated germ cell-specific genes *UTF1* and *FGFR3* were maintained until day 14 in SSC and SM cultures and until day 7 in PS cultures. This was in agreement with the previous human [12] and marmoset [19] culture studies using SSC media. In contrast to previous human culture studies [12] which were terminated at day 10, in the current study both *UTF1* and *FGFR3* expressing population were maintained at least until day 14 in SSC media indicating efficient maintenance of undifferentiated germ cell population. Similar to *UTF1* and *FGFR3* expression patterns, no significant change in expression levels of *MAGEA4*, pre- and early meiotic marker genes was observed until day 14 in all three culture conditions. Expression levels were significantly decreased at day 21 compared to day 0 in SSC media. *MAGEA4* expression levels were not significantly different in PS and SM media at day 21 compared to day 0. In congruence with diminishing Sertoli cell (*SOX9)* expression and early germ cell (*UTF1*, *FGFR3*, *MAGEAA4)* marker expression beyond day 14, decreased expression levels for differentiating germ cell markers *BOLL* and *DDX4* were observed during the culture period compared to day 0. The impact of the somatic cell ratio on germ cell survival, maintenance and expansion in cultures has not been systematically investigated and compared in previous primate testicular culture studies. However, limited evidence from previous human germ and somatic cell co-culture studies also demonstrate the effects of Sertoli cells on germ cell survival in culture. For instance, upregulation of expression of Sertoli cell marker *SOX9* coincided with maintenance of expression of pre-meiotic marker *FGFR3* during the 14-day culture period [18]. In contrast, upregulated expression of differentiating germ cell marker *DDX4* was observed only until day 7 [18]. In another human co-culture study, comparable patterns were observed as depletion of SOX9 expression in cultured cells coincided with depleting DDX4 expression [17].

Quantification of cultured cells expressing MAGEA4 was performed to understand the change in dynamics of the spermatogonial population in the three culture conditions during the culture period. MAGEA4 is known to be expressed by the spermatogonial cell population (A_dark_, A_pale_ and B spermatogonia) and dim by early pachytene spermatocytes in humans and marmosets [12,13,19]. Previous spermatogonial expansion *in vivo* studies show that spermatogenesis in macaques originates by A_pale_ spermatogonial divisions occurring at stage VII of the cycle of seminiferous epithelium [33]. Clusters of 2–4 spermatogonial cells are considered A_pale_ spermatogonia. Subsequently with further divisions cluster size increases with B1 spermatogonia containing 4–8 cell clusters, B2 spermatogonia containing 8–16 cell clusters, B3 comprising of 16–32 cell clusters and B4 consists of aggregated cluster of 32–64 cells [33]. Evidence from current cell quantification analysis substantiates the maintenance of small clusters expressing MAGEA4 in cultures indicating continued presence of an A_pale_ spermatogonial population in the cultures. In general, regardless of the culture conditions no significant changes were observed in overall population dynamics of MAGEA4-positive cultured cells overtime. The overall pattern of MAGEA4-expressing population dynamics for all the three culture conditions was comparable, indicating no significant influence of the culture media, serum and feeder-free culture conditions on macaque germ cell survival *in vitro*.

Our findings from germ cell expression and quantification data indicate that irrespective of the culture media used, the germ cells in culture are maintained until day 14 and lost thereafter. Gene expression data shows that diminishing expression levels of germ cell markers coincide with the diminishing Sertoli cell population in culture; however other somatic markers were expressed throughout culture period. To further validate and strengthen this finding, additional Sertoli cell marker could be characterized for macaque species and included in the marker panel for analysis in future studies. Nevertheless, the evidence obtained from the gene expression analysis employing eight marker genes in the current study leads us to speculate about a possible interdependence of these two specific testicular subpopulations (germ cells and Sertoli cells) for survival in culture. Previous testicular culture studies employed strategies of pre-sorting cellular sub-populations to investigate germ cell dynamics [44,45]. In contrast, the culture strategy and germ cell quantification approach employed to investigate the *in vitro* macaque germ cell dynamics in the current study, can be used as an efficient tool to follow up on the proliferation behavior of germ cells *in vitro*. The probable role of various components of testicular microenvironment influencing germ cell dynamics and expansion in culture should be further investigated in future studies.

This is the first study which focused on characterization and establishing macaque testicular primary cell culture to assesses change in germ cell expression levels and germ cell-somatic cell dynamics *in vitro*. Our findings demonstrate the effect of somatic components, specifically Sertoli cell survival on the maintenance of germ cell subpopulations in macaque germ cell-somatic cell co-culture overtime. Maintenance of undifferentiated macaque germ cells until day 14 in culture provides proof that macaques could be used as a research model for establishing *in vitro* germ cell-somatic cell cultures. These cultures could further be used to optimize culture conditions and investigate the effect of somatic microenvironment on spermatogonial regulation and germ cell maintenance for a prolonged period in culture. In addition, it provides new perspectives to study expansion mechanisms of primate spermatogonia in culture.

## Supporting information

S1 Fig Characterization of somatic cell and germ cell markers in neonatal macaque testicular sections. Representative images in the micrographs show strong expression for somatic cell markers SOX9 (A), VIM (B) and SMA (C) in neonatal macaque testicular sections. Intense expression of germ cell markers UTF1 (D), MAGEA4 (E) and VASA (F) was observed in neonatal macaque testicular sections; representative images have been shown in the lower panel. Black arrowheads in the micrographs indicate marker-specific localization. Scale bar in A, C, D, E and F indicates 20 μm, and in B indicates 50 μm.
(TIF)

S2 Fig Representative images for morphological observations of cultured adult macaque testicular cells at day 7 in SSC media condition. Cell morphology of cultured supernatant (SN) and attached (AT) fractions of macaque testicular cells at day 7 are represented in A and B respectively.
(TIF)

S3 Fig Gene expression levels of somatic marker genes *SOX9, VIM* and *ACTA2* relative to reference gene *GAPDH* of macaque testicular cells cultured in SSC, PS and SM media conditions for 21 days. Values are represented as mean ± SEM. Statistical significance (P<0.05 and P<0.02) is represented by single and double asterisk symbols (*, **) respectively.
(TIF)

S4 Fig Gene expression levels of germ cell marker genes *UTF1*, *FGFR3*, *MAGEA4*, *BOLL* and *DDX4* relative to reference gene *GAPDH* of macaque testicular cells cultured in SSC, PS and SM media conditions for 21 days. Significant differences (P<0.05 and P<0.02) are represented by single and double asterisk symbols (*, **) respectively.
(TIF)

S1 Table Details of Taqman assays used for RT-PCR analysis. (TIF)

S2 Table Details of antibodies used for immunohistochemistry and immunofluorescence analysis. (TIF)
