# Supplementary figures and images for: Characterization and population dynamics of germ cells in adult macaque testicular cultures
Source: PLoS One. 2019 Jun 21;14(6):e0218194. doi: 10.1371/journal.pone.0218194 (PMC6588212; doi:10.1371/journal.pone.0218194)

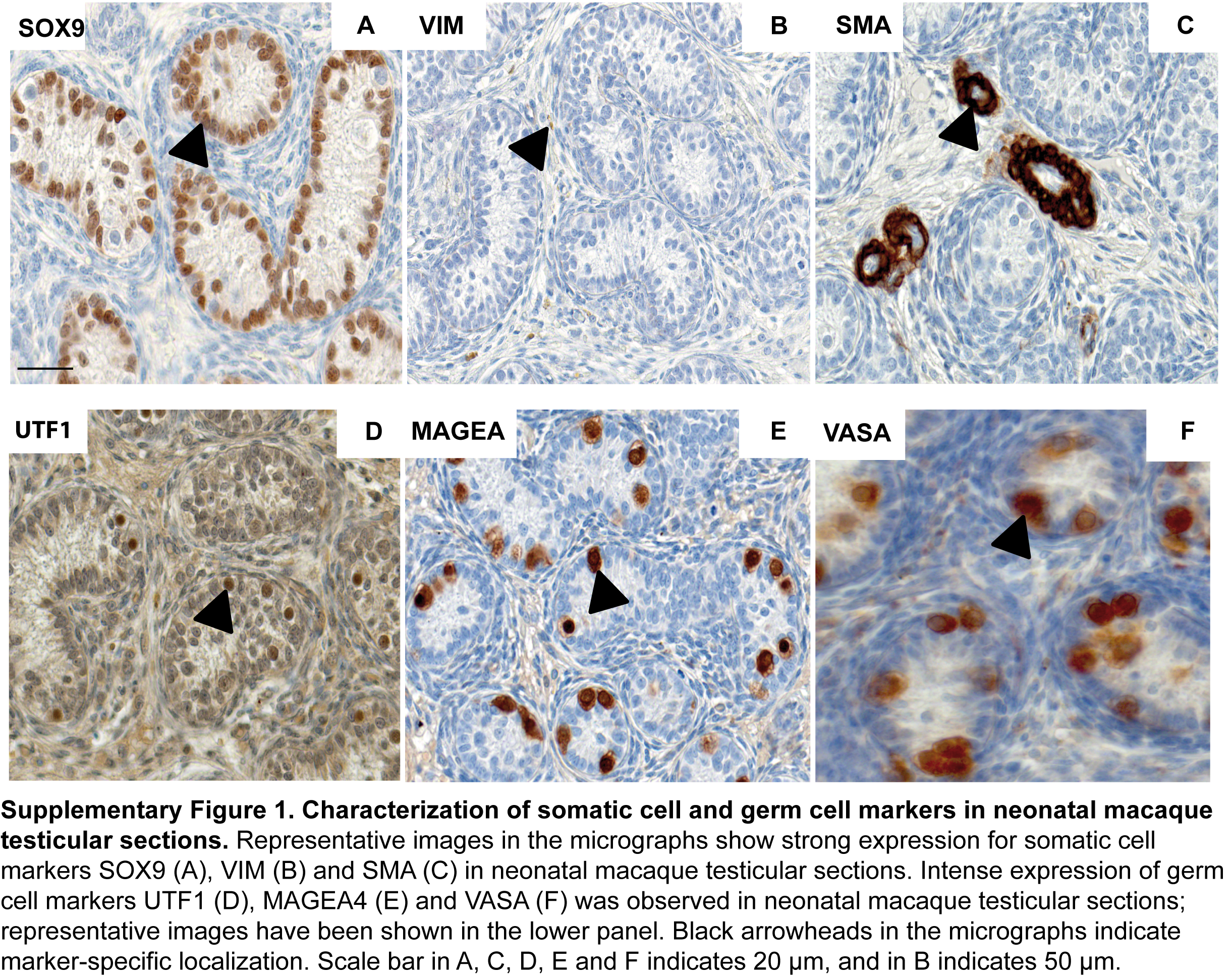

Supplement: S1 Fig — Representative images in the micrographs show strong expression for somatic cell markers SOX9 (A), VIM (B) and SMA (C) in neonatal macaque testicular sections. Intense expression of germ cell markers UTF1 (D), MAGEA4 (E) and VASA (F) was observed in neonatal macaque testicular sections; representative images have been shown in the lower panel. Black arrowheads in the micrographs indicate marker-specific localization. Scale bar in A, C, D, E and F indicates 20 μm, and in B indicates 50 μm. (TIF) [file pone.0218194.s001.tif]

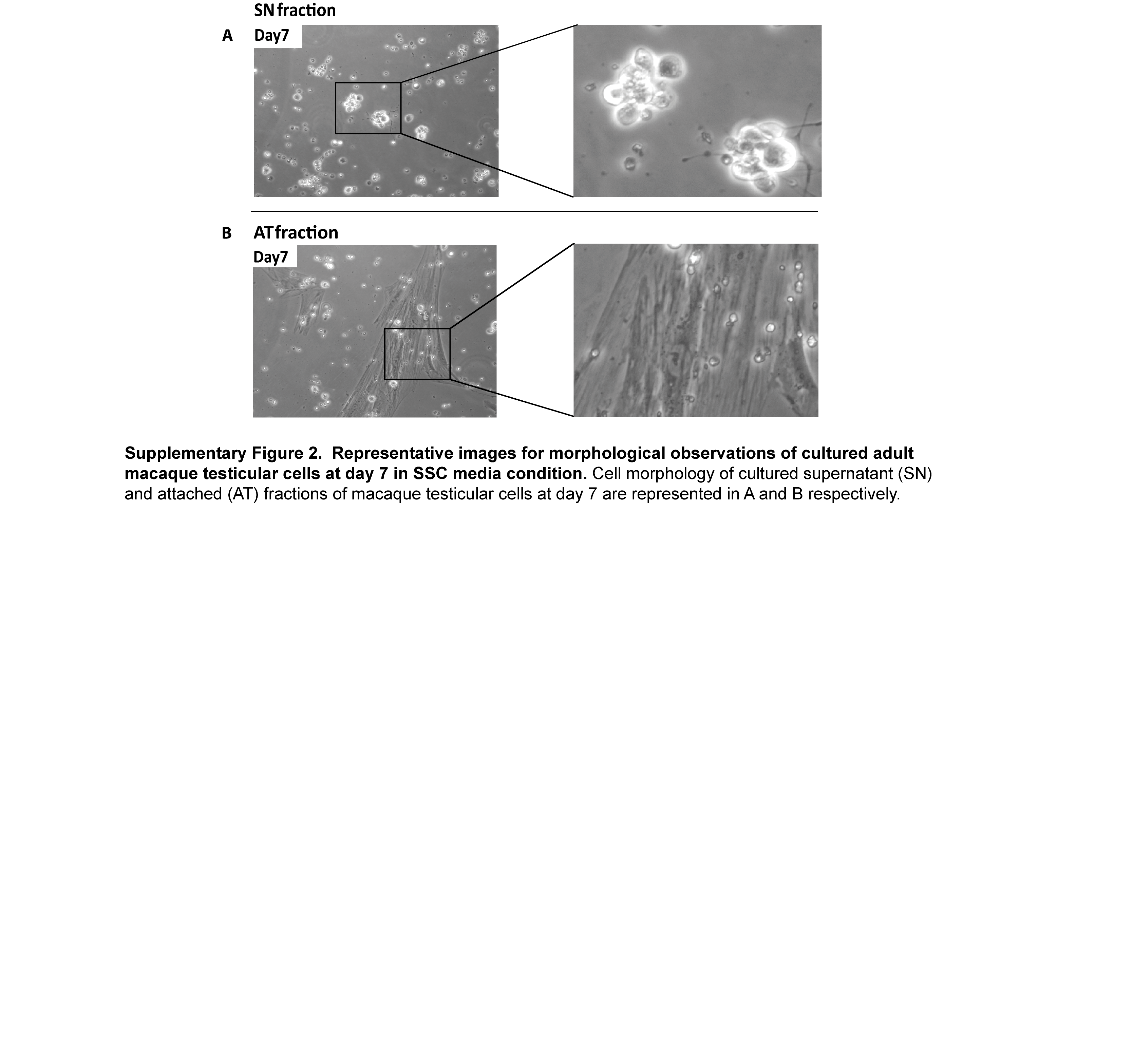

Supplement: S2 Fig — Cell morphology of cultured supernatant (SN) and attached (AT) fractions of macaque testicular cells at day 7 are represented in A and B respectively. (TIF) [file pone.0218194.s002.tif]

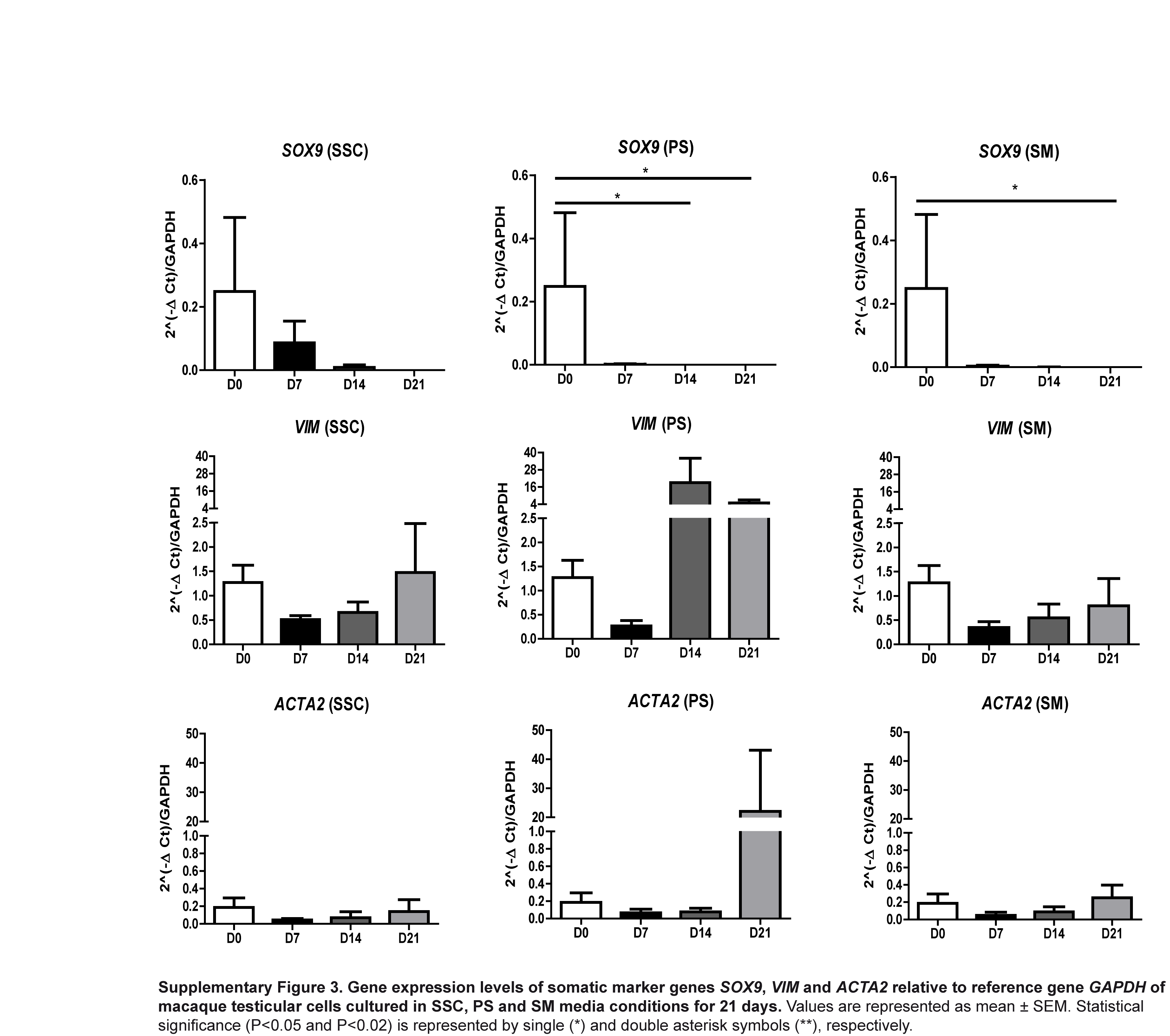

Supplement: S3 Fig — Values are represented as mean ± SEM. Statistical significance (P<0.05 and P<0.02) is represented by single and double asterisk symbols (*, **) respectively. (TIF) [file pone.0218194.s003.tif]

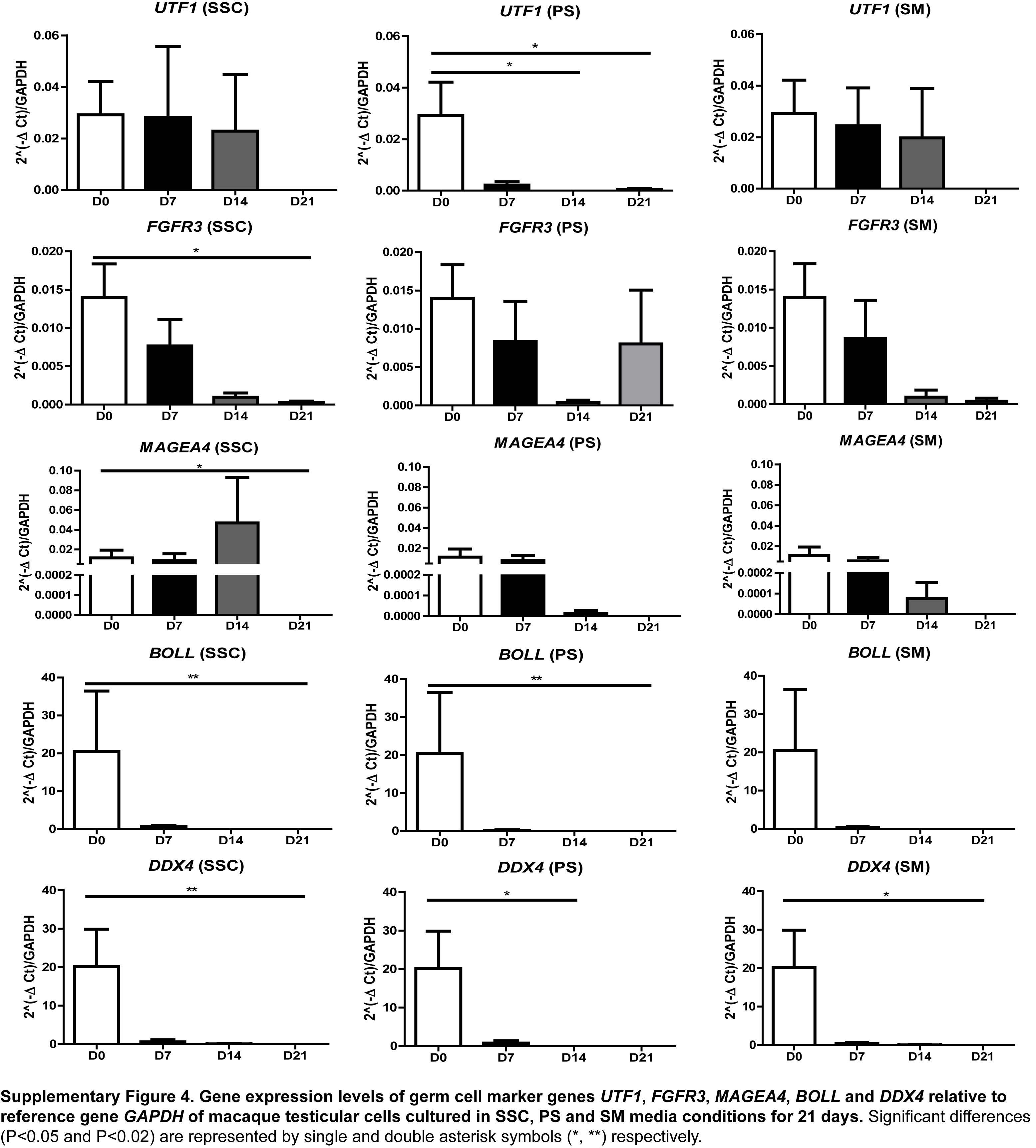

Supplement: S4 Fig — Significant differences (P<0.05 and P<0.02) are represented by single and double asterisk symbols (*, **) respectively. (TIF) [file pone.0218194.s004.tif]

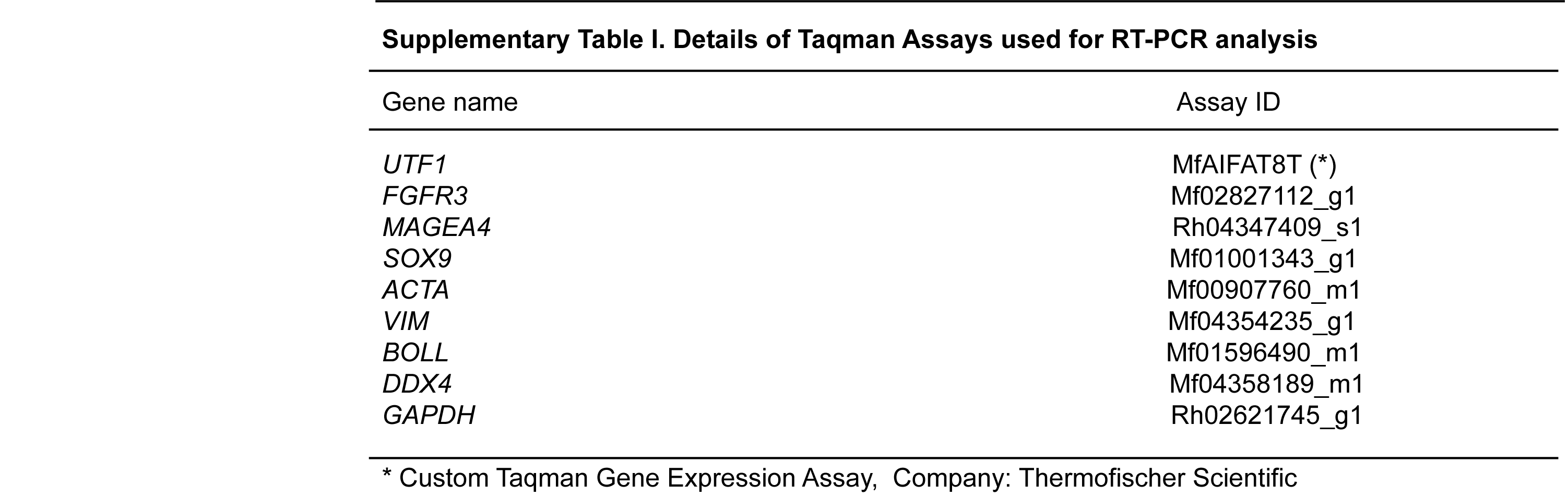

Supplement: S1 Table — (TIF) [file pone.0218194.s005.tif]

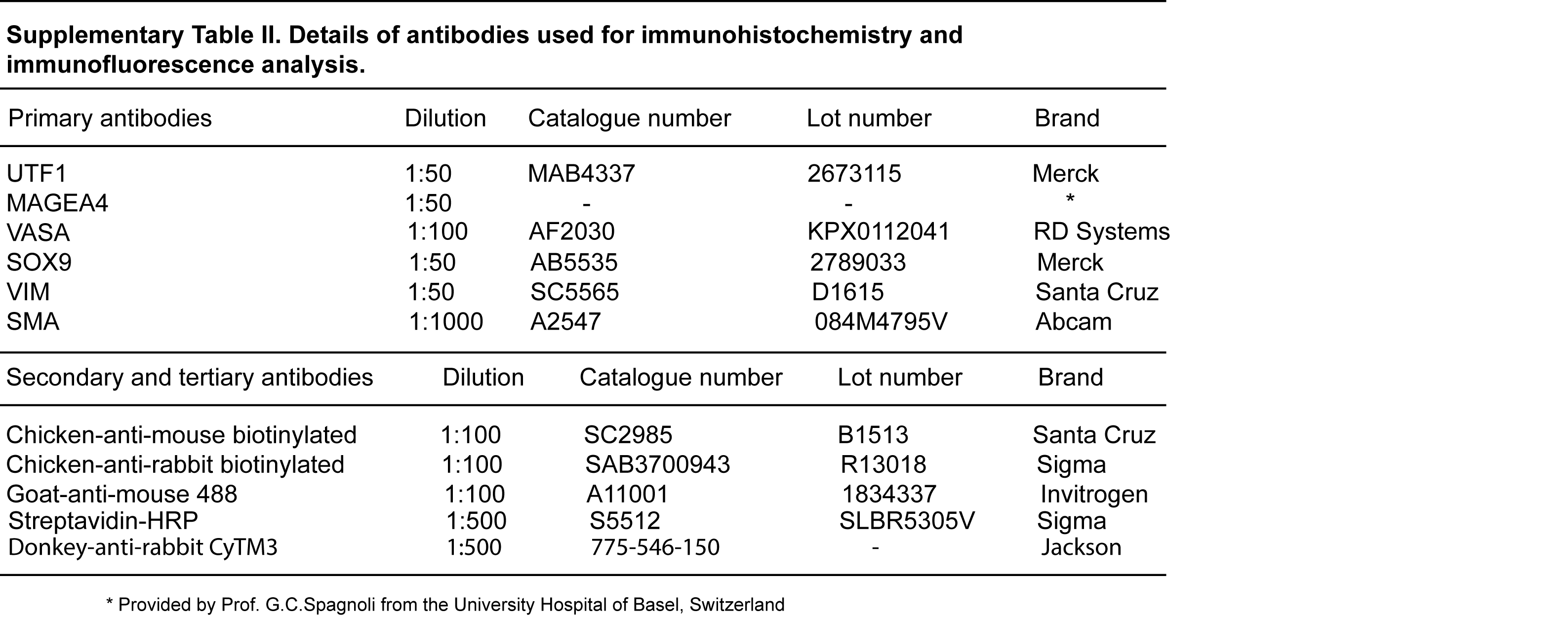

Supplement: S2 Table — (TIF) [file pone.0218194.s006.tif]
